# The Climatic Treatment of Consumption

**Published:** 1887-12

**Authors:** 


*The Climatic Treatment of Consumption.* By J. A. LINDSAY, M.A., M.D. Pp. 228. London: MACMILLAN & CO. 1887.

Probably no one ever sat down to write a work on Medical Climatology with a fuller personal experience of his subject than the author; and certainly no writer, so far as we know, has given to the profession a more useful book on the Climatic Treatment of Consumption. We have been charmed with the work. Simply as a book of travel, it may be read with unflagging interest from beginning to end. As a guide to the home practitioner, it supplies just that sort of knowledge which he wants to help him, telling in a plain, simple, and straightforward manner the practical advantages and drawbacks of every resort and every mode of travel. There is but little of theory—we have plenty of that from arm-chair travellers; and not much of that species of fact aggregated in meteorological columns; but plenty of hard sound sense as to warmth and cold, drought and moisture, food and clothing. We most heartily commend the book as being the best with which we are acquainted on the subject with which it deals.

